# [Corrigendum] High glucose and high insulin conditions promote MCF‑7 cell proliferation and invasion by upregulating IRS1 and activating the Ras/Raf/ERK pathway

**DOI:** 10.3892/mmr.2023.12967

**Published:** 2023-02-21

**Authors:** Mei-Lin Wei, Peng Duan, Zhi-Ming Wang, Miao Ding, Ping Tu

Mol Med Rep 16: 6690–6696, 2017; DOI: 10.3892/mmr.2017.7420

Subsequently to the publication of this paper, an interested reader drew to the authors’ attention that, in the wound-healing assays portrayed in Fig. 2A on p. 6692, in the 0 h row, the ‘NG + LI’ and ‘HG + HI’ panels contained overlapping data, such that they appeared to have been derived from the same original source.

After having examined their original data, the authors have realized that this figure was inadvertently assembled incorrectly. The corrected version of Fig. 2. showing the correct data for the ‘HG + HI’ panel, is shown on the next page. Note that this error did not significantly affect the results or the conclusions reported in this paper, and all the authors agree with the publication of this Corrigendum. Furthermore, the authors apologize to the readership for any inconvenience caused.

## Figures and Tables

**Figure 2.**
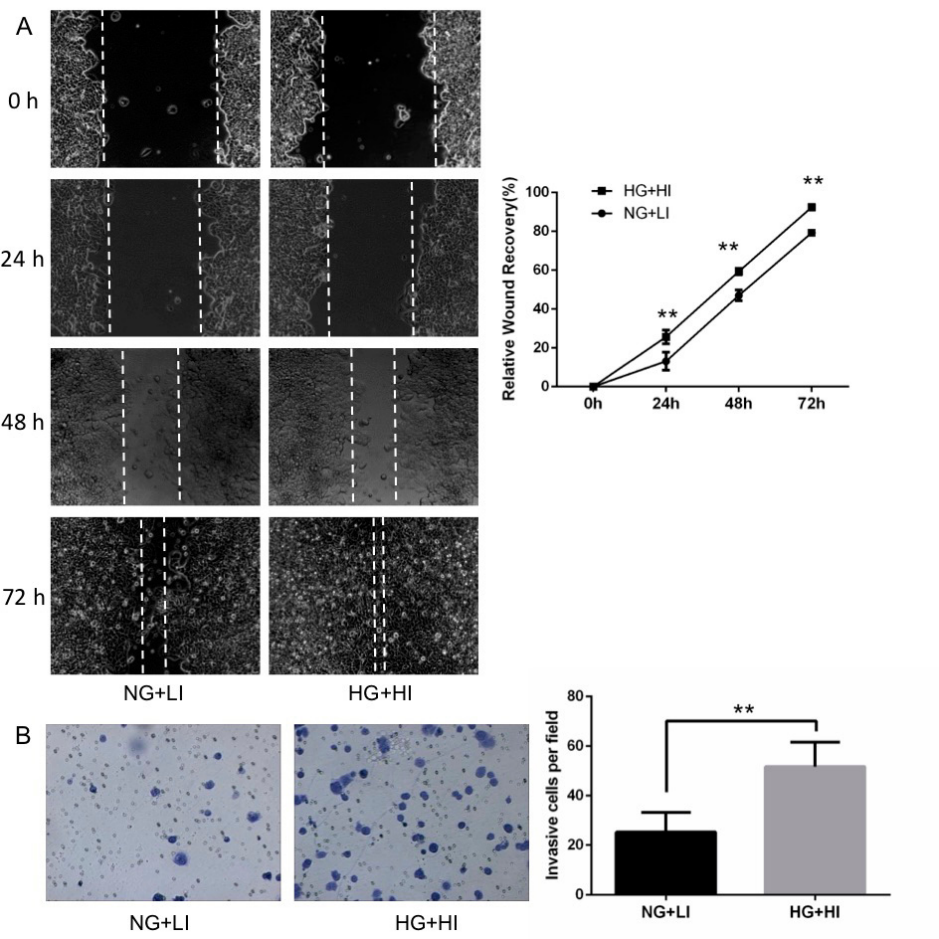
Effect of high glucose and high insulin on MCF-7 cell migration and invasion. (A) Migration ability was measured by wound healing assay and relative wound recovery was presented as % recovery of the wound distance at 24, 48 and 72 h relative to 0 h (magnification, ×100). (B) Invasion ability was assessed using transwell invasion assays (magnification, ×400). **P<0.01. NG+LI: Normal glucose + low insulin; HG+HI: High glucose + high insulin.

